# Addition of External Organic Carbon and Native Soil Organic Carbon Decomposition: A Meta-Analysis

**DOI:** 10.1371/journal.pone.0054779

**Published:** 2013-02-06

**Authors:** Weidong Zhang, Xiaofeng Wang, Silong Wang

**Affiliations:** 1 Huitong Experimental Station of Forest Ecology, State Key Laboratory of Forest and Soil Ecology, Institute of Applied Ecology, Chinese Academy of Sciences, Shenyang, PR China; 2 Huitong National Research Station of Forest Ecosystem, Huitong, PR China; 3 Graduate School of Chinese Academy of Sciences, Beijing, China; Utrecht University, The Netherlands

## Abstract

**Background:**

Extensive studies have been conducted to evaluate the effect of external organic Carbon on native soil organic carbon (SOC) decomposition. However, the direction and extent of this effect reported by different authors is inconsistent.

**Objective:**

The objective was to provide a synthesis of existing data that comprehensively and quantitatively evaluates how the soil chemical properties and incubation conditions interact with additional external organic C to affect the native SOC decomposition.

**Data Source:**

A meta-analysis was conducted on previously published empirical studies that examined the effect of the addition of external organic carbon on the native SOC decomposition through isotopic techniques.

**Results and Conclusions:**

The addition of external organic C, when averaged across all studies, enhanced the native SOC decomposition by 26.5%. The soil with higher SOC content and fine texture showed significantly higher priming effects, whereas the soil with higher total nitrogen content showed an opposite trend. The soils with higher C:N ratios had significantly stronger priming effects than those with low C:N ratios. The decomposition of native SOC was significantly enhanced more at early stage of incubation (<15d) than at the later stages (>15d). In addition, the incubation temperature and the addition rate of organic matter significantly influenced the native SOC decomposition in response to the addition of external organic C.

## Introduction

Soil organic carbon (SOC) decomposition is directly linked to atmospheric CO_2_ emissions, and consequently, to global climate change [1], [2]. Thus, extensive studies have been conducted to quantify, model, and predict the CO_2_ decomposition rate across a range of ecosystem types. Microbial activity, environmental variables (e.g., soil temperature, moisture) [3], [4], the initial soil chemical properties, and SOC inputs (e.g., plant litter, dead fine roots, and root exudates) generally influence the decomposition dynamics. When the inputs are not considered, SOC decomposition depends on SOC quality [5], microbial activity, and temperature. In all ecosystems, plant litter, dead fine roots, and root exudates serve as SOC inputs and influence its output through their priming effect (PE) [6]. PEs are defined as short-term changes in the turnover of SOC caused by the addition of external organic C [7]. A positive PE accelerates the decomposition of the native SOC, whereas a negative PE retards it. Numerous studies have been performed on the PE through the addition of ^13^C- or ^14^C-labeled plant materials [8]–[10] or easily degraded C sources [11], [12] to simulate the input of organic C that occurs in natural ecosystems. However, the direction of the PEs reported by different authors is inconsistent. Several studies have shown that the native SOC decomposition is significantly enhanced by the addition of external organic C [13]. However, others have reported either a suppression effect or no significant change [14]–[16]. The possible mechanisms of PEs have been suggested by previous studies [7], [17], but the relationship between the SOC decomposition and external organic C is still unclear. Consequently, the direction and extent of the PE is still unpredictable.

The different responses of native SOC decomposition to the addition of external organic C across various studies can be partially explained by differences in soil chemical properties. The PE is driven by soil microorganisms [18]; thus, the PE of soil with higher SOC, which often indicates a higher soil microbial biomass [19], is activated more by the external organic C. As suggested by Kuzyakov, the PEs in soil with higher SOC content are stronger than those in poor soil [7]. While the SOC content clearly influences the extent of priming effect induced by external organic C, other factors, including both nutrient status and incubation conditions, may be important in explaining the large difference in the extent of priming effects observed in the literature.

Meta-analysis quantitatively assesses the evidence for or against a particular hypothesis, which is advantageous over narrative or qualitative reviews that lack sampling rigor and robust statistical methods [20]. Meta-analysis methodology has been used widely by ecologists to analyze the response of soil respiration and aboveground plant growth to experimental ecosystem warming [21], the effect of forest fires on soil microbial biomass and N mineralization [22], and the response of crop productivity to bio-char application [23]. Although several authors have reviewed the studies in our analysis and the possible mechanisms of the PEs, to our knowledge, no meta-analysis has been done on this topic.

The objective of the current meta-analysis is to provide a comprehensive and quantitative synthesis of the effect of external organic C on native SOC decomposition. This paper evaluates how soil chemical properties (e.g., SOC content, total nitrogen content, and C:N ratio) and the incubation conditions (i.e., substrate quality, incubation stage, incubation temperature, and addition rate) influence the response of the native SOC decomposition to the addition of external organic C.

## Methods

### Data collection

More than 250 studies on SOC decomposition published since 1980 were reviewed and studies wherein the external organic C was experimentally manipulated under laboratory conditions were identified (22 studies and some unpublished data, Table S1). In many of these studies, the effects of environmental factors such as added nitrogen, temperature, and soil moisture on the soil organic carbon decomposition were evaluated. This meta-analysis focused on the influence of additional external organic C on native soil organic carbon decomposition. The database for the meta-analysis was created according to the following criteria: First, the experimental studies reported direct manipulation of the soil through the addition of external organic C in the form of plant material and other small molecules, and had to assess the response in terms of the CO_2_ release dynamics. Second, the CO_2_ production from organic C by the native soil system can be separated by ^13^C- or ^14^C-labeled technique, and the native SOC decomposition rate can be measured directly. Third, sufficient information regarding environmental variables, such as native soil chemical properties and experimental conditions, are provided in the studies. For example, a large number of the studies that examined the relationship between live roots and the soil reported native soil respiration using isotopic techniques [24]–[27]. However, the rate of root exudates secretion is unquantifiable and experimental conditions such as temperature were not provided. Thus, studies those examined the effects of living plant on native SOC decomposition were all excluded from the current analysis.

The native SOC decomposition rate is expressed as mg C kg^−1^ soil h^−1^. In some studies, the results were displayed as the CO_2_ production accumulation dynamics during the incubation stage. The SOC decomposition rate and its variance were estimated using the following equations:
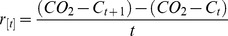


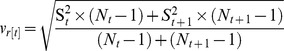
where *CO_2_ – C_t+1_* and *CO_2_ – C_t_* are the means of the accumulative CO_2_ production derived from native soil at time *t* and *t*+1, respectively; *S_t_* and *S_t_*_+1_ are the standard deviations of the accumulative CO_2_ production derived from native soil at time *t* and *t*+1, respectively; *N_t_* and *N_t_*_+1_ are the number of replicates for the respective times; and *t* is the sampling time during incubation. The Origin 7.0 software with the “Digitize. OPK” plug-in was used to extract data from the figures.

In addition to the native SOC decomposition rate, the data on the related environmental variables in these studies were also collected, including the soil chemical properties (SOC content, total nitrogen content, C:N ratio, and soil texture) and the experimental conditions (substrate quality, incubation stage, incubation temperature, and addition rate). The SOC content ranged from 6 g kg^−1^ to 340 g kg^−1^. The total nitrogen content ranged from 0.63 g kg^−1^ to 26.8 g kg^−1^. The C:N ratio ranged from 0.63 to 38.1. The addition rate ranged from 0.1% to 33.3% of SOC. The incubation temperature ranged from 6.5°C to 36.5°C. More than 15 different organic C types were represented in the database. The incubation stage was monitored for periods ranging from <1 d to 98 d.

### Meta-analysis

The strength of the effect for each paired observation was calculated as the natural log of the response ratio *R*  =  *x_t_*/*x_c_*, where *x_t_* is the mean of the added organic C for the treatment and *x_c_* is the mean of the associated control without the addition of organic C. The variance of ln *R* was estimated using the following equation: *v*_ln*R*_  =  *s_t_* ^2^/(*N_t_* × *X_t_* ^2^) + *s_c_* ^2^/(*N_c_* × *x_c_* ^2^) [28]. The standard methods in the software MetaWin (version 2.0) were used to perform the analyses. The procedure is analogous to the ANOVA, wherein the total heterogeneity (*Q_t_*) for a group of comparisons is partitioned into the within-group (*Q_w_*) and between-group (*Q_b_*) heterogeneity. The *Q_w_* is tested against a chi-square distribution with *m* – 1 degrees of freedom, where *m* is the number of groups.

In addition to examining the overall effect of organic C addition on native soil organic carbon decomposition, this study also determined whether the chemistry of the native soil and the experimental conditions elicited a significantly different response to the addition of organic C. We hypothesized that the response of the native soil organic carbon decomposition to the addition of organic C is related to the properties of native soil. Therefore, the combined data were grouped according to the chemistry of native soil. The studies were categorized based on the soil organic carbon content (<20 g kg^−1^ or >20 g kg^−1^), the total nitrogen content (<2 g kg^−1^ or >2 g kg^−1^), the C:N ratio (<10 or >10) and soil texture (Coarse, Medium and Fine texture). Furthermore, the effects of the experimental conditions on the response of the native SOC decomposition to organic C were also determined. Generally speaking, small-molecule substrates such as fructose, glucose and amino acids are easy to be utilized by microorganisms, and defined as high-quality substrates in the current analysis. Otherwise, plant materials, such as maize, slurry, and wheat straw with lower microbial availability are defined as low-quality substrates. The data were also grouped according to the substrate quality of the added organic C (Low or High), the incubation stage (<15 or >15 d), the incubation temperature (≤20, 20–25, or >25°C), and the addition rate in terms of the % SOC (<4% or >4%). That information can be found in Table S2. Besides, matrix style table was provided in Table S3 to show which different substrates were used in which soil types (according to soil C and N content). The heterogeneity among the categorical groups was partitioned to determine the effect of each category (e.g., incubation temperature) on the response of the native SOC decomposition to the addition of organic C. The *Q_b_* for all categorical variables was tested at a significance level of 0.05. Then, the mean effect strength for each category was calculated, with the 95% confidence intervals (CIs) generated using the bias-corrected bootstrap procedure in the MetaWin software. The cumulative effect of all studies or each categorical group was considered significant if its 95% CI did not reach zero, and the cumulative effect is significantly different between two categorical groups if their 95% CIs were non-overlapping [29].

## Results and Discussion

**Figure 1:**
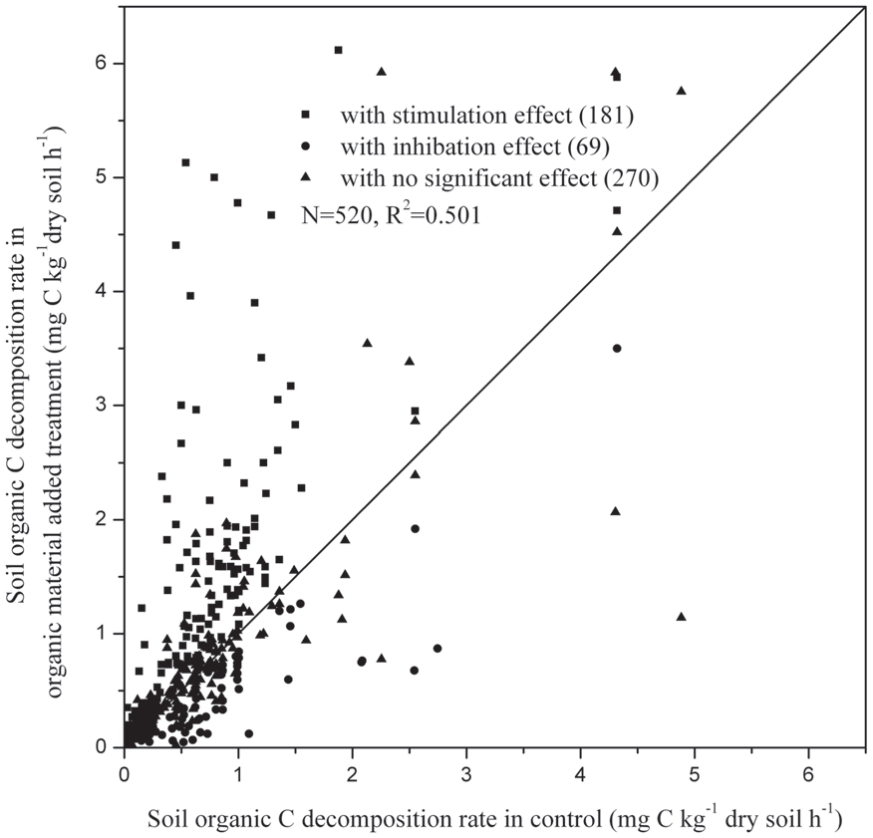
Relationship between soil organic C decomposition rates in organic material treatment and in control. Each point represents a single comparison between the external organic C addition and control treatment. Values falling on the 1:1 line indicate a similar decay response for organic C addition vs control treatments, whereas points above or below the line indicate a stimulation or inhibition in decomposition, respectively.

**Figure 2:**
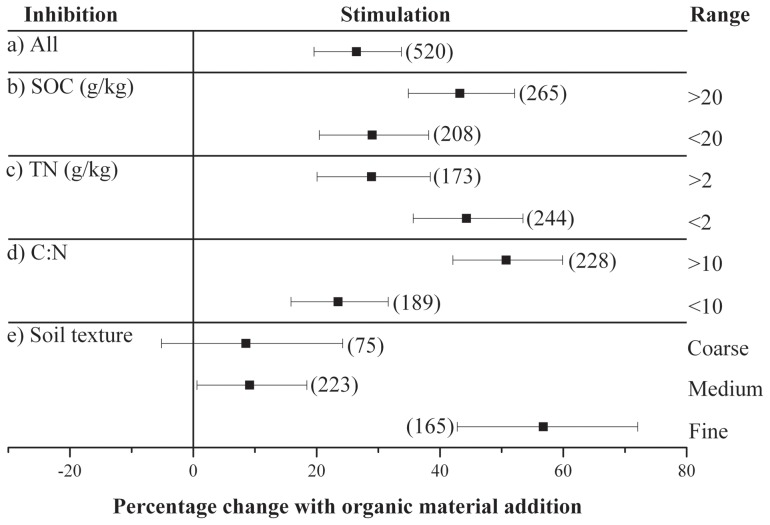
Response of soil organic C decomposition to external organic C addition when the data were grouped by (b) soil organic C content, (c) soil total N content, (d) the C:N ratio in soil and (e) soil texture. The number of effect-size comparisons for each response variable is shown in parentheses.

This meta-analysis is the first quantitative synthesis of published literature describing the native SOC decomposition in response to the addition of external organic C. A large number of studies have examined the effects of external organic C addition on native SOC decomposition, but little is known about the extent of the native SOC response. Of the 520 observations in the meta-analysis, 69 showed significant inhibition effects, 181 showed significant stimulation effects, and 270 showed no statistically significant effects. Only 50.1% of the heterogeneity of the native SOC decomposition rates after the addition of organic C can be explained by the decomposition rates in the associated controls (Fig. 1). The analysis showed that the addition of external organic C enhances the native SOC decomposition by 26.5% (Fig. 2). However, the amplitude of the native SOC decomposition response was very large (ranged from 95.1% inhibition to 1207% stimulation). Previous studies have suggested mechanisms for the so-called PEs. Generally speaking, positive priming effects were induced due to microbial growth and the accompanying increased enzyme production; possible negative priming mechanisms include the toxicity of the substrates to microorganisms and preference uptake of C-rich substrates by microorganisms [7]. However, the complicated interaction of the external organic C and the native SOC has made predicting the effect of the addition of external organic C difficult. The response of the native SOC decomposition largely depends on the chemical properties of the soil and the experimental conditions.

The results from the between-group heterogeneity analysis showed that the response of native SOC decomposition to external organic C addition depends on the chemical properties of the soil, including the SOC content, TN content, C:N ratio, and soil texture (Table 1). The SOC content in the current analysis ranged from 6 g kg^−1^ to 340 g kg^−1^. Soil with a higher SOC content generally has higher decomposition rates, which can be confirmed by the observed SOC decomposition rate of the controls. The CO_2_ release rate in soil with higher SOC content (>20 g kg^−1^) was 0.80 mg C kg^−1^ soil h^−1^, which is significantly higher than that of soil with low SOC content (0.22 mg C kg^−1^ soil h^−1^). Additionally, the SOC content also influenced the strength of the PE. The analysis showed that the addition of organic C significantly enhances the decomposition of native SOC with low (<20 g kg^−1^) and high (>20 g kg^−1^) SOC content by 29.0% and 43.2%, respectively. This result is consistent with that of Kuzyakov, who suggested that the PEs in soils rich in C are more intense than those in poor soils [7].

**Table 1:** Effect of organic material additions on between-group heterogeneity (*Q*_b_) for soil organic C decomposition rate.

Categorical variable	k	Q_b_
Soil organic C (g/kg)^a^	472	5.1*
Soil total N (g/kg)^a^	416	5.6*
C:N ratio^a^	416	20.5**
Soil texture^a^	462	38.0**
Material quality (Low or High)^b^	519	0.02ns
Incubation time (days)^b^	519	4.6*
Incubation temperature (°C)^b^	519	67.5**
Material added (as % soil C)^b^	472	5.6*

* P<0.05; **P<0.01.

K: the number of mean comparisons within each level of analysis.

a: categories were <20 and >20 g kg^−1^ for SOC, <2 and >2 g kg^−1^ for nitrogen content, <10 and >10 for C:N ratio, Fine, Medium and Coarse for soil texture.

b: categories were Low and High for material quality, <15d and >15d for incubation time, <4 and >4% soil C for addition rate, ≤20°C, 20–25°C and >25°C for incubation temperature.

Several studies were excluded because the level of soil organic C, soil total N was not available in those studies.

The PE strength also depends on the soil N content (Table 1), which ranged from 0.63 g⋅kg^−1^ to 26.8 g⋅kg^−1^ in this analysis. Soils with higher N content exhibited higher SOC decomposition rates in the controls. However, the response of PE to N content was contrary to that of SOC decomposition. The overall PEs in soil with N content below 2 g⋅kg^−1^ was 44.3%, significantly higher than that in soils with higher N content (28.9%). A similar response pattern was also observed for the C:N ratio. These phenomena are likely related to the limitation in the soil microbial nutrient. Microorganisms generally require C and other nutrients at characteristic stoichiometric ratios. In soils with lower N content and higher C:N ratios, the microorganism activity caused by organic C addition involves the scavenging of N from native SOM, resulting in more intense PEs.

The manner by which soil texture might influence the extent of priming effects has not been paid considerable attention. In this study, we observed significant between-group heterogeneity (Qb) among soils with different soil textures (Table 1). Soils with fine texture have significantly higher priming effect (56.7%) than soils with medium and coarse textures (Fig. 2). This phenomenon could be attributed to the low N content and high C:N ratio of these soils. In this case, microorganism activity caused by organic C addition decomposed the native SOM more intensely to obtain available N for growth, resulting in the higher priming effects. However, the difference in the priming effects between soils with medium (9.1%) and coarse (8.5%) textures was not significant. The data in the coarse texture category were derived from only three studies, which caused large variation within groups (Fig. 2). Thus, another factor other than soil texture may account for the observed extent of priming effect.

**Figure 3:**
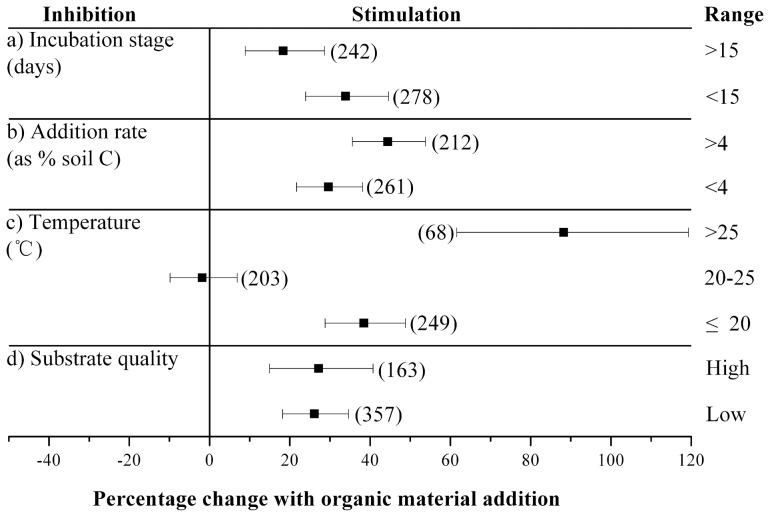
Response of soil organic C decomposition to external organic C addition when the data were grouped by (a) incubation stage, (b) organic material addition rate (expressed as % of soil C) (c) incubation temperature, (d) organic C quality. The number of effect-size comparisons for each response variable is shown in parentheses.

The response of native SOC decomposition to organic substrates addition also depended on experimental conditions (Table 1; Fig. 3). PE strength changed because of the amount of added C and energy, as suggested by Blagodatskaya and Kuzyakov [17]. A higher rate of organic C addition likely activates more microorganisms, resulting in a stronger PE. SOC content differed among various studies. Hence, the amount of added substrate C was presented as percentage of SOC. In this analysis, significant between-group heterogeneity was observed among soils that received different levels of additional organic C (Table 1). Corresponding to the percentage of additional organic C to the SOC content, soils that received more substrate C showed stronger PE (44.4%) than soils that received less substrate C (29.6%). However, most studies on PE consisted of no more than three addition rates. Strong evidence on the relationship between the strength of PEs and addition rate of organic C is still lacking. The minimum addition rate of organic C that induces a PE and the maximum PE that occurs when excess external organic C is added into soil remain unknown. Studies involving more addition rates are still needed to clarify this relationship further.

PEs are defined as “short-term changes in the turnover of soil organic matter caused by comparatively moderate treatments of the soil” [7]. The duration of this PEs remains unknown. Perelo and Munch [30] observed significant positive PE that lasted for more than 80 d after white mustard residues were added into the soil. However, whether PEs has commonly long durations remain undetermined. The present analysis suggests that PE strength is influenced by the incubation stage (Fig. 3). The extent of PEs (33.9%) during the early stage of incubation (<15 d) was significantly higher than that (18.4%) during the later stage (>15 d). This result is reasonable considering that the organic substrates in the soil decreased during incubation as a result of decomposition. Previous studies have shown that the extent of PE decrease with incubation [31], and this meta-analysis validated this finding.

The external organic C involved in the current analysis included low-quality plant materials (e.g., maize, wheat, and slurry) and high-quality small molecules (e.g., fructose, alanine, oxalic acid, roots exudates, etc.). Given that high-quality substrates have more available organic C for the microorganisms than low-quality substrates, high-quality organic C is likely to induce higher PE than the low-quality organic C. However, between-group heterogeneity was not significant in terms of organic C quality (Table 1). We attribute this discrepancy to two conditions. First, although plant materials are categorized as low quality, they also contain large amounts of small molecules that are highly available for microorganisms. Second, the addition rate of organic C in the low-quality group was much higher than that in the high-quality group (13.66% of SOC vs. 2.85% of SOC), and the amount of added C is important in determining the extent of PE as discussed above.

The response of priming effect to temperature has seldom been studied, because most studies on priming effect used only one incubation temperature. In this study, we observed significant between-group heterogeneity among different incubation temperature ranges. As suggested by Kuzyakov [6], the PE below 20°C (38.4%) was significantly more intense than above 20°C (−0.17%). In addition, based on short-term incubation (24 h), we found that the extent of the PEs induced by both glucose and Chinese fir litter in two soils (natural forest and Chinese fir plantation soils) consistently decreased along the temperature gradient (unpublished data), consistent with Kuzyakov's prediction [6]. One possible explanation for this phenomenon is the higher native SOC decomposition level in the controls under higher temperatures. In this case, native SOC decomposition level hardly increased further through substrate addition, which resulted in lower PE. However, the extent of PE above 25°C (88.2%) was much higher than that below 25°C. We attribute this inconsistent trend to other factors in the current analysis. For example, this group only contained 68 observations and applied considerably more organic substrate (11.1% of SOC). The relationship between temperature and PE is evidently important in predicting the response of SOC decomposition in future warming, and related research is fairly insufficient. Further studies are needed to examine directly temperature sensitivity of PE with other factors.

In summary, the results indicate that addition of external organic C alters the native SOC decomposition, but the direction and extent of response are mediated by interaction between native soil chemical properties and incubation conditions. Native SOC decomposition was enhanced under the following conditions: SOC content higher than 20 g⋅kg^−1^; total N content below 2 g⋅kg^−1^; C:N ratio higher than 10 and soils with fine texture. In addition, PE was significantly stronger during the early incubation stage (<15 d) and at higher organic C addition rates. This meta-analysis indicated the importance of organic C input on soil carbon cycling in ecosystems. The strength of this effect is closely related to the native soil chemical properties and environmental conditions. Moreover, given that microbes drive the priming effect, different soils with diverse microbial communities may respond to substrate addition differently. However, information on the influence of microbial community in native soil on priming effect is very limited, and this phenomenon requires further studies.

## Supporting Information

Table S1 **The 23 studies included in the meta-analysis database, along with addition rate, incubation temperature and the type of external organic C.** Full citations follow the table.
(DOCX)

Table S2 **Studies within each subdivision of the categorical variables.**
(DOCX)

Table S3 **Substrates and soils used in the published papers.**
(DOCX)
