# Supplementary material for: Addition of External Organic Carbon and Native Soil Organic Carbon Decomposition: A Meta-Analysis
Source: PLoS One. 2013 Feb 6;8(2):e54779. doi: 10.1371/journal.pone.0054779 (PMC3566129; doi:10.1371/journal.pone.0054779)
Supplement: Table S1 — The 23 studies included in the meta-analysis database, along with addition rate, incubation temperature and the type of external organic C. Full citations follow the table. (DOCX) [file pone.0054779.s001.docx]

Table S1. The 23 studies included in the meta-analysis database, along with addition rate, incubation temperature and the type of external organic C. Full citations follow the table.

| Reference | Addition rate  (as % of SOC) | Incubation temperature (℃) | Substrate type |
| --- | --- | --- | --- |
| 1 Hamer & Marschner (2005) | 1.33 | 20 | Fructose, Alanine, Oxalic acid, Catechol |
| 2 Hartley et al. (2010) | ND | 10 | Glucose, Glycine |
| 3 Bol et al. (2003) | 33.3 | 27 | Slurry |
| 4 Magid et al. (1999) | 5.3 | 14 | *Lolium perenne* residue |
| 5 Marx et al. (2007) | 9, 10 | 14 | Maize and wheat exudates |
| 6 Hoyle et al. (2008) | 0.17 | 25 | Glucose |
| 7 Luna-Guido et al. (2003) | 1.1, 1.6, 1.8, 2.2, 3.1, 3.5 | 22 | Maize |
| 8 Bell et al. (2003) | ND | 25 | Wheat |
| 9 Blagodatskaya et al. (2007) | 0.1, 9.7 | 22 | Glucose |
| 10 Fangueiro et al., (2007) | 33.3 | 25 | Slurry |
| 11 Nottingham et al. (2009) | 15 | 20 | Sugar, Maize litter |
| 12 Fontaine et al. (2004) | 4.8 | 28 | Cellulose |
| 13 Garcia-Pausas & Paterson (2011) | 0.8 | 20 | Glucose |
| 14 Perelo & Munch (2005) | 2.9, 3.6 | 14 | *Sinapis alba* residue, Glucose |
| 15 Chotte et al. (1997) | 5.3 | 28 | Glucose, Starch, Legume, Wheat |
| 16 Kuzyakov & Bol (2006) | 0.3, 0.15 | 27 | Slurry, Sugar |
| 17 Aoyama et al. (2000) | 2.6 | 25 | Glucose |
| 18 Falchini et al. (2003) | 0.18 | 25 | Glucose, Oxalic acid, Glutamic acid |
| 19 Guenet et al. (2012) | 15.5, 1.5, 17, 1.7 | 20 | Cellulose, Straw |
| 20 Zhang & Wang (2012) | 11.8 | 16.5 | Chinese fir, Alder |
| 21 Fontaine et al. (2011) | 3.1 | 20 | Cellulose |
| 22 Garcia-Pausas & Paterson (2011) | 6.4 | 20 | Glucose |
| 23 Unpublished data | 5 | 6.5, 16.5, 26.5, 36.5 | Chinese fir, Glucose |

1 Hamer U, Marschner B (2005) Priming effects in different soil types induced by fructose, alanine, oxalic acid and catechol addition. Soil Biol Biochem 37: 445-454.

2 Hartley IP, Hopkins DW, Sommerkorn M, Wookey PA (2010) The response of organic matter mineralization to nutrient and substrate additions in sub-arctic soils. Soil Biol Biochem 42: 92-100.

3 Bol R, Moering J, Kuzyakov Y, Amelung W (2003) Quantification of priming and CO_2_ respiration sources following slurry-C incorporation into two grassland soils with different C content. Rapid Commun Mass Sp 17: 2585-2590.

4 Magid J, Kjaergaard C, Gorissen A, Kuikman PJ (1999) Drying and rewetting of a loamy sand soil did not increase the turnover of native organic matter, but retarded the decomposition of added ^14^C-labelled plant material. Soil Biol Biochem 31: 595-602.

5 Marx M, Buegger F, Gattinger A, Zsolany Ả, Munch JC (2007) Determination of the fate of ^13^C labeled maize and wheat exudates in an agricultural soil during a short-term incubation. Eur J Soil Sci 58: 1175-1185.

6 Hoyle FC, Murphy DV, Brookes PC (2008) Microbial response to the addition of glucose in low-fertility soils. Biol Fertil Soils 44: 571-579.

7 Luna-Guido ML, Vega-Estrada J, Ponce-Mendoza A, Hernandez-Hernandez H, Montes-Horcasitas MC, et al. (2003) Mineralization of ^14^C-labelled maize in alkaline saline soils. Plant Soil 250: 29-38.

8 Bell JM, Smith JL, Bailey VL, Bolton H (2003) Priming effect and C storage in semi-arid no-till spring crop rotations. Biol Fertil Soils 37: 237-244.

9 Blagodatskaya EV, Blagodatsky SA, Anderson TH, Kuzyakov Y (2007) Priming effects in Chernozem induced by glucose and N in relation to microbial growth strategies. Appl Soil Ecol 37: 95-105.

10 Fangueiro D, Chadwick D, Dixon L, Bol R (2007) Quantification of priming and CO_2_ emission sources following the application of different slurry particle size fractions to a grassland soil. Soil Biol Biochem 39: 2608-2620.

11 Nottingham AT, Griffiths H, Chamberlain PM, Stott AW, Tanner EVJ (2009). Soil priming by sugar and leaf-litter substrates: A link to microbial groups. Appl Soil Ecol 42: 183-190.

12 Fontaine S, Bardoux G, Benest D, Verdier B, Mariotti A, et al. (2004) Mechanisms of the priming effect in a Savannah soil amended with Cellulose. Soil Sci Soc Am J 68: 125-131.

13 Garcia-Pausas J, Paterson E (2011) Microbial community abundance and structure are determinants of soil organic matter mineralization in the presence of labile carbon. Soil Biol Biochem 43: 1705-1713.

14 Perelo LW, Munch JC (2005) Microbial immobilization and turnover of ^13^C labeled substrates in two arable soils under field and laboratory conditions. Soil Biol Biochem 37: 2263-2272.

15 Chotte JL, Ladd JN, Amato M (1997) Sites of microbial assimilation, and turnover of soluble and particulate ^14^C-labelled substrates decomposing in a clay soil. Soil Biol Biochem 30: 205-218.

16 Kuzyakov Y, Bol R (2006) Sources and mechanisms of priming effect induced in two grassland soils amended with slurry and sugar. Soil Biol Biochem 38: 747-758.

17 Aoyama M, Angers DA, N’Dayegamiye A, Bissonnette N (2000) Metabolism of ^13^C-labeled glucose in aggregates from soils with manure application. Soil Biol Biochem 32: 295-300.

18 Falchini L, Naumova N, Kuikman PJ, Bloem J, Nannipieri P (2003) CO_2_ evolution and denaturing gradient gel electrophoresis profiles of bacterial communities in soil following addition of low molecular weight substrates to simulate root exudation. Soil Biol Biochem 36: 775-782.

19 Guenet B, Juarez S, Bardoux G, Abbadie L, Chenu C (2012) Evidence that stable C is as vulnerable to priming effect as is more labile C in soil. Soil Biol Biochem 52: 43-48.

20 Zhang WD, Wang SL (2012) Effects of NH_4_^+^ and NO_3_^-^ on litter and soil organic carbon decomposition in a Chinese fir plantation forest in South china. Soil Biol Biochem 47: 116-122.

21 Fontaine S, Henault C, Aamor A, Bdioui N, Bloor JMG, et al. (2011) Fungi mediate long term sequestration of carbon and nitrogen in soil through their priming effect. Soil Biol Biochem 43: 86-96.

22 Garcia-Pausas J, Paterson E (2011) Microbial community abundance and structure are determinants of soil organic matter mineralisation in the presence of labile carbon. Soil Biol Biochem 43: 1705-1713.
