# Supplementary material for: Addition of External Organic Carbon and Native Soil Organic Carbon Decomposition: A Meta-Analysis
Source: PLoS One. 2013 Feb 6;8(2):e54779. doi: 10.1371/journal.pone.0054779 (PMC3566129; doi:10.1371/journal.pone.0054779)
Supplement: Table S2 — Studies within each subdivision of the categorical variables. (DOCX) [file pone.0054779.s002.docx]

Table S2. Studies within each subdivision of the categorical variables.

| Categorical variable | Studies within each categorical subdivision |
| --- | --- |
| SOC content (g kg^-1^) |  |
| <20 | 1, 3, 4, 5, 6, 8, 10, 12, 14, 15, 16, 18, 19, 20 |
| >20 | 1, 3,7,9,11,16,17, 21, 22, 23 |
| Nitrogen content (g kg^-1^) |  |
| <2 | 5, 7, 8,11, 12, 14,18, 19, 20, 23 |
| >2 | 1, 3, 9, 10, 16, 17, 21, 23 |
| C:N ratio |  |
| <10 | 1, 3, 5, 10, 14, 16, 18, 21 |
| >10 | 1, 7, 9, 11, 12, 14, 17, 19, 20, 23 |
| Substrate quality |  |
| High | 1,2,5,6,9,11, 12,13,14,15,16,17, 18, 22, 23 |
| Low | 3,4,7,8,10,11,14,15, 19, 20, 21, 23 |
| Incubation stage (days) |  |
| <15 day | 1, 2, 3, 4, 5, 6, 7, 9, 10, 11, 12, 13, 14, 15, 16, 17, 18, 19, 20, 21, 22, 23 |
| >15 day | 3, 4, 5, 6, 7, 8, 10, 11, 12, 14, 15, 19, 20, 21, 22 |
| Incubation temperature (℃) |  |
| ≤20 | 1, 2, 4, 5, 11, 13, 14, 19, 20, 21, 22, 23 |
| 20-25 | 6, 7, 8, 9, 10, 17,18 |
| >25 | 3,12,15,16, 23 |
| Addition rate (as % of SOC) |  |
| <4 | 1, 6, 7, 9, 13, 14, 16, 17, 18, 19, 21 |
| >4 | 3, 4, 5, 7, 9, 10, 11, 12, 14, 15, 20, 22, 23 |
