# Supplementary material for: Addition of External Organic Carbon and Native Soil Organic Carbon Decomposition: A Meta-Analysis
Source: PLoS One. 2013 Feb 6;8(2):e54779. doi: 10.1371/journal.pone.0054779 (PMC3566129; doi:10.1371/journal.pone.0054779)
Supplement: Table S3 — Substrates and soils used in the published papers. (DOCX) [file pone.0054779.s003.docx]

Table S3. Substrates and soils used in the published papers

|  | SOC < 20 g kg^-1^ | | |  | SOC > 20 g kg^-1^ | | |  | ND |
| --- | --- | --- | --- | --- | --- | --- | --- | --- | --- |
|  | < 2 g kg^-1^ | > 2 g kg^-1^ | ND |  | < 2 g kg^-1^ | > 2 g kg^-1^ | ND |  |  |
| Alanine |  | 1 |  |  |  | 1 |  |  |  |
| Catechol |  | 1 |  |  |  | 1 |  |  |  |
| Fructose |  | 1 |  |  |  | 1 |  |  |  |
| Glucose | 14, 18 |  | 6, 22 |  | 23 | 9, 17, 23 | 13 |  | 2 |
| Glutamic acid | 18 |  |  |  |  |  |  |  |  |
| Glycine |  |  |  |  |  |  |  |  | 2 |
| Oxalic acid | 18 | 1 |  |  |  | 1 |  |  |  |
| Root exudates | 5 |  |  |  |  |  |  |  |  |
| Sugar |  | 16 |  |  | 11 | 16 |  |  |  |
| Alder | 20 |  |  |  |  |  |  |  |  |
| Cellulose | 12, 19 |  |  |  |  | 21 |  |  |  |
| Chinese fir | 20 |  |  |  | 23 | 23 |  |  |  |
| Legume |  |  | 15 |  |  |  |  |  |  |
| *Lolium perenne* |  |  | 4 |  |  |  |  |  |  |
| Maize |  |  |  |  | 7, 11 |  |  |  |  |
| *Sinapis alba* | 14 |  |  |  |  |  |  |  |  |
| Slurry |  | 10, 16 |  |  |  | 3 |  |  |  |
| Starch |  |  | 15 |  |  |  |  |  |  |
| Wheat |  |  | 15 |  |  |  |  |  | 8 |
| Straw | 19, 30 |  |  |  |  |  |  |  |  |
